# Slip estimation model for traversability-based motion planning of cargo rover on extraterrestrial surface

**DOI:** 10.3389/frobt.2025.1638667

**Published:** 2025-11-05

**Authors:** Taisei Nishishita, Genya Ishigami

**Affiliations:** Graduate School of Integrated Design Engineering, Faculty of Science and Technology, Keio University, Yokohama, Japan

**Keywords:** terramechanics, motion planning, traversability, slip estimation, SCM, project chrono

## Abstract

As part of the robotics technologies required for *In-situ* resource utilization (ISRU), the development of cargo rovers for transporting resources is needed. However, these cargo rovers have unique technical challenges that differ from conventional exploration rovers, including the need to traverse rough terrains with their varying mass due to transporting payloads. Moreover, research addressing these challenges has been limited, and the relevant technologies have not been fully established. To address these challenges, this paper proposes a parametric model for estimating wheel slippage. The model is formulated as a function of four input parameters: slope angle, rover heading angle, payload mass, and wheel angular velocity, and is applicable to resource-transporting rovers with varying mass. Additionally, the use of a parametric model reduces computational load, which offers advantages for onboard implementation. The proposed estimation model was quantitatively evaluated by comparing datasets obtained from multi-body dynamics analysis. This paper also introduces a new traversability assessment model which incorporates the proposed slip estimation model. We demonstrated the proposed model by integrating it into a sampling based motion planning. The simulation result of the motion planning show that the planner with our model can generate safer motions and enables the rover to reach the target regardless of the cargo payload.

## 1 Introduction

In recent years, there has been active discussion regarding long-term exploration activities of planets and moons. The Artemis program, for example, aims to establish technologies for sustainable human presence in space, with the Moon as a base for future Mars missions. A key component of Artemis is *In-Situ* Resource Utilization (ISRU), which seeks to use local resources for producing water, fuel, and construction materials (Smith et al., 2020; Sanders and Kleinhenz, 2024; Werkheiser et al., 2024). Achieving ISRU will require advanced robotics, particularly rovers capable of transporting resources to designated locations. Additionally, future operations may involve multiple rovers, and there is an increasing expectation for rovers to move autonomously while considering traversability. However, implementing resource-transporting rovers presents several technical challenges not encountered with conventional exploration rovers. First, there are differences in the environmental conditions in which rovers are operated. One of the most critical risks in rover operations is the vehicle getting stuck in sand, which is extremely difficult to recover from via remote control, and in some cases, this could directly lead to mission failure. Conventional exploration rovers have been operated conservatively, avoiding areas prone to slipping, due to the unique circumstances of space missions, such as being one-time missions and the fact that human repairs are not feasible. However, when considering applications for resource transportation, the roads in excavation areas are unpaved, and repeated rover movements and excavation activities can alter the terrain, impeding the rover mobility. Autonomous systems capable of safely operating in such harsh environments have not yet been demonstrated in space, making this a highly challenging technical task. Next, transporting resources means that the rover’s weight varies dynamically. The mass properties are closely linked to the rover dynamics, making the rover traversability significantly varies.

Experiments have been conducted to investigate the traversability of rovers, revealing that slip characteristics are influenced by a wide range of factors (Ishigami et al., 2007; Chhaniyara et al., 2012; Sutoh et al., 2010). Based on the result, numerous non-parametric models have been proposed for estimating slip characteristics (Sevastopoulos and Konstantopoulos, 2022). Non-parametric models have the advantage of being able to flexibly represent complex real-world phenomena. Gonzalez et al. (2018) proposed a machine learning algorithm to estimate slip characteristics using proprioceptive sensors such as RTK-GPS and IMUs, without relying on environmental information such as terrain slope. A major advantage of this method, which solely uses proprioceptive sensors, is that it does not require computationally expensive processes like Visual Odometry and is not affected by environmental conditions such as optical conditions. Another end-to-end approach involves a neural network model that estimates slip ratio from three parameters: translational speed, slope angle, and heading angle (Sakayori and Ishigami, 2021). This model is designed to output both the slip ratio and energy consumption simultaneously. One key feature of neural network models is their ability to integrate various types of information into a single architecture for estimation. However, as the phenomena become more complex and the dimensionality of the model increases, a large amount of training data is required. This raises concerns, especially in the context of planetary exploration, about whether sufficient datasets can be prepared. Furthermore, the low interpretability of black-box models can lead to issues such as overfitting. Consequently, ensuring that these models do not exhibit unexpected behavior requires exhaustive validation across a wide range of scenarios, which makes their application to real missions challenging.

The main contributions of this paper are as follows:

• A new parametric slip estimation model that incorporates parameters such as mass variation and wheel velocity.
• A new traversability assessment model that integrates the above slip estimation model.

In this paper, we propose a new slip estimation model using parametric representation. The model is not only interpretable, but is formulated based on terramechanics theory to minimize deviation from the actual environment. The impact of rover mass on slip characteristics is also considered, and incorporates this factor into the model. As a result, the model is expected to be used for applications where mass variations are anticipated, such as construction and resource transport rovers on the lunar surface. Moreover, by formulating the model in a parametric manner, the computational load is reduced compared to non-parametric machine learning-based methods. This advantage facilitates implementation on space-grade CPUs with limited computational resources. Additionally, we propose a new traversability assessment model that uses the developed slip model. This model incorporates uncertainties, such as model errors, into its parameters. We further demonstrate the motion planning that explicitly considers the slip risk using the proposed model.

The outline of this paper is as follows. In Section 2, we propose a parametric slip estimation model. Multi-body dynamics simulations were conducted to identify the key factors influencing slip, and the identified factors were then incorporated into the proposed model. Section 3 introduces the traversability assessment model incorporating the proposed parametric models. Section 4 presents the results of simulation verification that the proposed risk metrics can be used to move to the target point while minimizing the risk of wheel slip.

## 2 Slip estimation model

### 2.1 Slip metrics definition

First, the concept for evaluating slippage is explained. When a rover traverses on loose soil, the wheels of a rover can slip, resulting in a difference between the desired and actual velocity vectors (Wong, 1978). Slip in the longitudinal direction can be quantified by the slip ratio *s*, as shown in Equation 1:

$$s=1-\frac{v_{x}}{v_{\text{ref}}},$$ (1)

where $v_{x}$ is the linear velocity in the longitudinal direction. The $v_{\text{ref}}$ is the desired velocity vector and is expressed as $v_{\text{ref}}=r\omega$ using the wheel radius r and the angular velocity of the wheel ω . When the wheel does not slip and the desired translational velocity is generated, s=0 , and when the wheel is stuck and the velocity is zero, s=1 . There are various definitions of lateral slip, but in this study, we use the slip angle as defined in Equation 2.

$$\beta =\tan^{-1}\left(\frac{v_{y}}{v_{x}}\right),$$ (2)

where $v_{y}$ is the linear velocity in the lateral direction. The slip angle is an index that expresses the degree of lateral velocity relative to longitudinal velocity in terms of an angle.

### 2.2 Parametric slip model

Slippage is closely related to various environmental and rover system conditions. When estimating slip ratios and slip angles, one approach would be to consider as many of these factors as possible and build nonparametric models using techniques such as machine learning. However, while onboard sensors such as LiDAR can measure the terrain geometry, it can hardly directly identify soil properties such as cohesion and friction. Therefore, in this study, we extract parameters that are relatively easy to measure and have a significant impact on slip metrics, and approximate them using a parametric model for computational simplicity. One advantage of the parametric model is that it reduces computational load.

**FIGURE 1:**
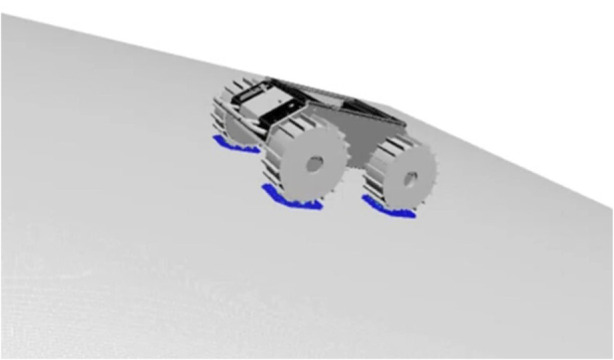
Multi-body dynamics simulation with project chrono.

Investigating the relationship between various parameters and slip characteristics through experiments is not practical due to the enormous number of test cases required and accurately reproducing space conditions on Earth is difficult. Therefore, simulating low-gravity conditions and analyzing rover behavior is also a valuable approach for space applications. In fact, studies have analyzed the slip characteristics of NASA’s VIPER using a multi-body dynamics simulator (Hu et al., 2025). In this study, the open-source software Project Chrono was used to create datasets (Tasora et al., 2016; Mazhar et al., 2013). Considering the trade-off between computation time and model accuracy, the Soil Contact Model (SCM) was adopted (Krenn and Hirzinger, 2009). SCM represents the wheel as a polygonal mesh and performs contact detection with the ground for each grid, calculating the pressure acting on the wheel based on the Bekker-Wong theory (Bekker, 1969). This calculation is very simple, light on computational load, because assuming ideal contact conditions. SCM provides results that are reasonably close to actual phenomena, but the soil on actual planetary surfaces may consist of mixed soils with different properties and may include coarse materials such as gravel and rocks. As another approach, Discrete Element Method (DEM) simulations provide results that are more accurate than SCM, but it is still known to deviate from real-world behavior. In addition, DEM requires long computation times, making it unsuitable for applications such as generating large datasets, as in this study. Figure 1 shows a simulation of a 4-wheeled rover in the Project Chrono environment. The area where the wheels are in contact with the soil and pressure is generated is visualized in different colors.

**FIGURE 2:**
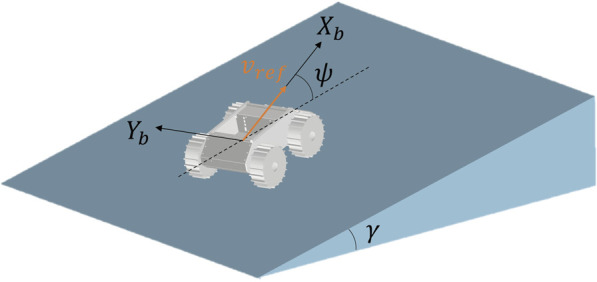
Definition of the slope angle γ , heading angle ψ and reference velocity $v_{\text{ref}}$ .

The parameter conditions for obtaining the dataset are shown in Table 1. The definitions of slope angle γ and heading angle ψ are illustrated in Figure 2. Slope angle, heading angle, total mass, and wheel angular velocity were selected as the parameters with particularly large effects on slippage, and data were collected for a total of 840 cases. The simulation was performed with parameter conditions within the range where the analysis was stable. For example, when the slope angle exceeds 25° or the translational velocity reaches 0.5 m/s, inertial and other dynamic effects become significant, causing the assumptions of the quasi-static motion model to break down and reducing the model’s reproducibility. However, such extreme parameter regions are not included in the applicable range of the slip risk metric introduced later in this study, because in practice, operation under these conditions would be excluded already at the motion planning stage. Therefore, these extreme conditions do not need to be explicitly considered for the purposes of this study. Constructing a dataset to evaluate the robustness of the proposed model is impractical because it would require handling a large number of parameters and an enormous number of analysis cases. Instead, it is more reasonable to assess robustness through experimental validation, which we consider as future work, while focusing here on the model’s nominal fitting ability. In addition, developing online parameter adaptation strategies for slip estimation, given the large parameter space, is a substantial challenge beyond the scope of this study and is also identified as future work. The slip ratio and slip angle are calculated based on measurements from each wheel and subsequently aggregated into representative values for the rover as a whole. The mass range was determined by assuming that the rover itself weighs 20 kg and that the payload can be up to 80 kg. In the case of a lunar rover application, the vehicle’s mass and payload are expected to be larger. However, the proposed slip estimation model described later is parametric, allowing it to be formulated in the same manner. Although the behavior should change depending on soil conditions, these parameters are not directly used to estimate the slip ratio, but rather other parameters are adjusted to simulate differences in characteristics depending on soil conditions.

**TABLE 1:** SCM simulation conditions.

Parameter	Range
Slope angle γ [degrees]	− 15, − 10, − 5, 0, 5, 10
Heading angle ψ [degrees]	0, 15, 30, 45, 60, 75, 90
Linear velocity $v_{x}$ [m/s]	0.05, 0.10, 0.15, 0.20
Total mass m [kg]	20, 40, 60, 80, 100

**FIGURE 3:**
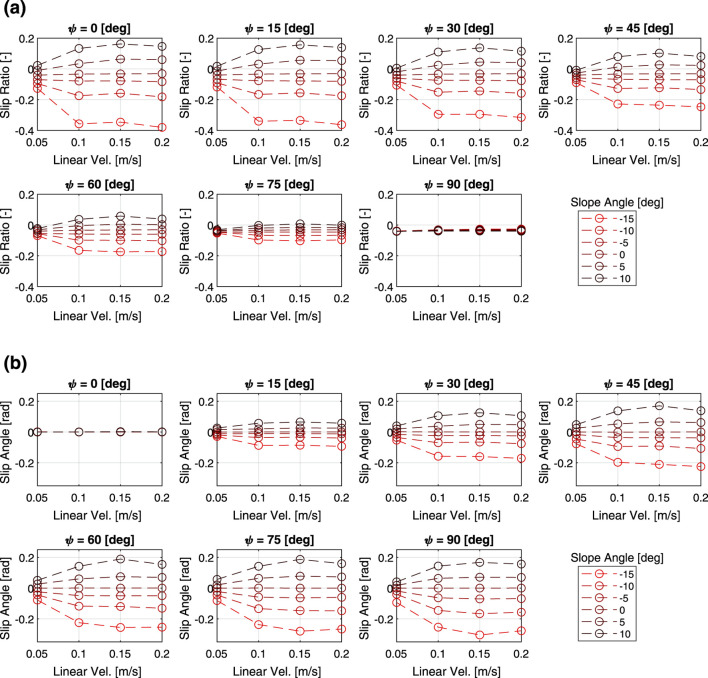
Multi-body dynamics simulation results: **(a)** slip ratio vs. linear velocity, **(b)** slip angle vs. linear velocity.

Figure 3 shows the relationship between the slip characteristics and the three parameters of heading angle, slope angle and linear velocity command. Here, the mass is fixed at 60 kg. Regardless of the other parameters, the slip ratio tends to increase as the slope angle increases. The slip ratio increases as the heading angle approaches zero, while the slip angle tends to decrease. The slip ratio and slip angle also tended to change more sensitively with the slope angle as the translational speed increased. In addition, while linear velocity had a large effect on the slippage when the velocity is small, the slippage do not change much when the velocity increases to a certain degree.

**FIGURE 4:**
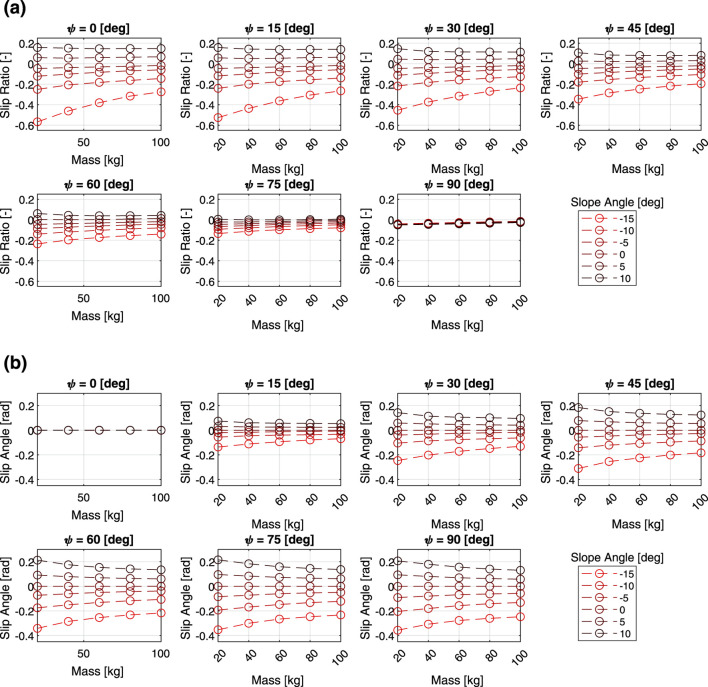
Multi-body dynamics simulation results: **(a)** slip ratio vs. total mass, **(b)** slip angle vs. total mass.

Figure 4 shows the results of the verification of how the slip characteristics change depending on the three parameters of heading angle, slope angle, and mass, with linear velocity fixed at 0.2 m/s. The same relationship between the slope angle, heading angle, and slip characteristics can be seen in the figures. As the mass decreases, the slip ratio becomes more sensitive to the slope angle. A similar trend was observed in the relationship between slip angle and mass.

Based on the trends of various parameters and slip characteristics obtained from the dataset, the results of formulating the slip ratio s and slip angle β as a parametric model are as follows.

$$s\left(\gamma ,\psi ,\omega ,k_{m}\right)=a_{1}\tan\left(a_{2}\gamma \right)\cdot \cos\left(\psi \right)\cdot \tanh\left(a_{3}\omega \right)\cdot e^{-a_{4}k_{m}}+a_{5},$$ (3)

$$\beta \left(\gamma ,\psi ,\omega ,k_{m}\right)=b_{1}\tan\left(b_{2}\gamma \right)\cdot \sin\left(\psi \right)\cdot \tanh\left(b_{3}\omega \right)\cdot e^{-b_{4}k_{m}}+b_{5},$$ (4)

where $a_{1}\ldots a_{5}$ and $b_{1}\ldots b_{5}$ are hyperparameters, which is tuned according to environmental conditions and rover configurations. $k_{m}$ is the ratio of the total mass to the reference mass $m_{0}$ . The slip ratio and slip angle have the same equations except where the effect of the heading angle is expressed. The influence of each parameter on slip characteristics is formulated individually, and the overall model is expressed as a product of these terms. For example, when the slope angle γ=0 , the rover is assumed to be traveling on flat terrain, and the first term on the right-hand side becomes zero. The proposed slip estimation model is constructed using a dataset generated by a multibody dynamics simulator that incorporates uncertainties such as rover system noises and environmental disturbances. As a result, the proposed model is formulated with explicit consideration of these uncertainties. In other words, rather than isolating and addressing each source of uncertainty individually, the proposed approach adopts a data-driven strategy to verify that the model functions robustly under the presence of various uncertainties. The following subsection qualitatively discusses the meaning of each term in the proposed slip estimation model.

### 2.3 Discussion on slip model

The parametric model of slip ratio and slip angle proposed in this study is discussed from a terramechanics perspective. Let $\mu_{s}$ be the coefficient of static friction, the conditions for the rover to slip against the ground are given by Equation 5

$$\mu_{s}>\tan\left(\gamma \right).$$ (5)

In other words, whether the rover slips or not is determined by the tangent of the slope angle. While the slip behavior on soft soil is not strictly deterministic, we simplify the model by assuming that the slip ratio and the slip angle, which indicate how prone the rover is to slipping, are proportional to tan⁡γ , as shown in the first factors of the first product terms in Equations 3, 4, respectively.

The direction of the drawbar pull that the rover is subjected to due to gravity is determined by the heading angle ψ . For example, when ψ=0 , the rover is facing upslope, and the slip ratio is the highest at this time. Since the drawbar pull force acting on the rover is proportional to cos(ψ) , the slip ratio is also modeled to be proportional to cos(ψ) , as shown in the second factor of the first product term in Equation 3.

**FIGURE 5:**
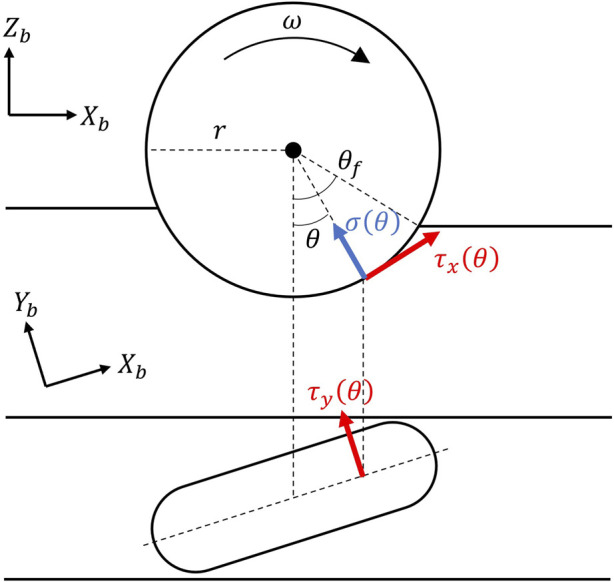
Wheel-soil contact model.

The normal and shear stresses beneath a wheel on the loose soil can be modeled as shown in Figure 5. In Equations 3, 4, the effect of angular velocity is formulated using hyperbolic tangents. If the angular velocity is sufficiently low, the rover behavior follows the terramechanics theory under static conditions. Equation 6 represents the shear stress τ (Janosi and Hanamoto, 1961).

$$\tau_{i}=\left(c+p\left(\theta \right)\tan\left(\varphi \right)\right)\left[1-e^{-j_{i}/k_{i}}\right]\;\;\left(i=x,y\right),$$ (6)

where c represents the cohesion stress, φ is the internal friction angle, $j_{x}$ and $j_{y}$ is the total soil deformation. p(θ) is the normal stress. $k_{x}$ and $k_{y}$ is the shear deformation modules. $j_{x}$ and $j_{y}$ are the total soil deformations and are expressed as follows (Wong and Reece, 1967; Yoshida and Ishigami, 2004):

$$j_{x}\left(\theta \right)=r\left[\theta_{f}-\theta -\left(1-s\right)\left(\sin\left(\theta_{f}\right)-\sin\left(\theta \right)\right)\right],$$ (7)

$$j_{y}\left(\theta \right)=r\left(1-s\right)\left(\theta_{f}-\theta \right)\cdot \tan\left(\beta \right),$$ (8)

where $\theta_{f}$ is the entry angle. As the angular velocity increases, the wheels move more soil, which can leads to an increase in j . Additionally, from Equations 8, 9, it is clear that as the slip ratio s increases, $j_{x}$ also increases. Therefore, the simulation results, which show that the slip ratio s increases with angular velocity, are consistent with this. Furthermore, $j_{y}$ increases as the slip angle β increases, indicating that as angular velocity increases, the slip angle β also increases. These characteristics are incorporated into the third factors in the first product terms of Equations 3, 4, respectively.

The fourth factors in the first product terms of Equations 3, 4 corresponds to the mass-dependent term. As the mass increases, the slip ratio becomes less sensitive to the slope angle, and a similar trend is observed for the slip angle. This is likely because the increased mass causes the wheels to sink into the ground, increasing the contact area between the wheel’s surface and the soil, which in turn increases the shear stress.

### 2.4 Hyperparameters optimization

In the previous section, the relationship between various parameters and slippage was investigated through multi-body dynamics simulations, and a new parametric models of slip ratio and slip angle were proposed. Here, we discuss the results of hyperparameter tuning to investigate how well the proposed model can simulate the simulation results. By solving the following optimization problem, the hyperparameters of the slip ratio model, $a≔\left[a_{1},\ldots ,a_{5}\right]$ are adjusted.

$$a^{*}=\underset{a}{\mathrm{arg min}}\underset{\gamma }{\sum }\underset{\psi }{\sum }\underset{\omega }{\sum }\underset{k_{m}}{\sum }{\left[s_{\text{SCM}}\left(\gamma ,\psi ,\omega ,k_{m}\right)-s\left(\gamma ,\psi ,\omega ,k_{m}\right)\right]}^{2}.$$ (9)

The hyperparameters of the slip angle model $b≔\left[b_{1},\ldots ,b_{5}\right]$ are obtained by solving the following equation.

$$b^{*}=\underset{b}{\mathrm{arg min}}\underset{\gamma }{\sum }\underset{\psi }{\sum }\underset{\omega }{\sum }\underset{k_{m}}{\sum }{\left[\beta_{\text{SCM}}\left(\gamma ,\psi ,\omega ,k_{m}\right)-\beta \left(\gamma ,\psi ,\omega ,k_{m}\right)\right]}^{2}.$$ (10)

**FIGURE 6:**
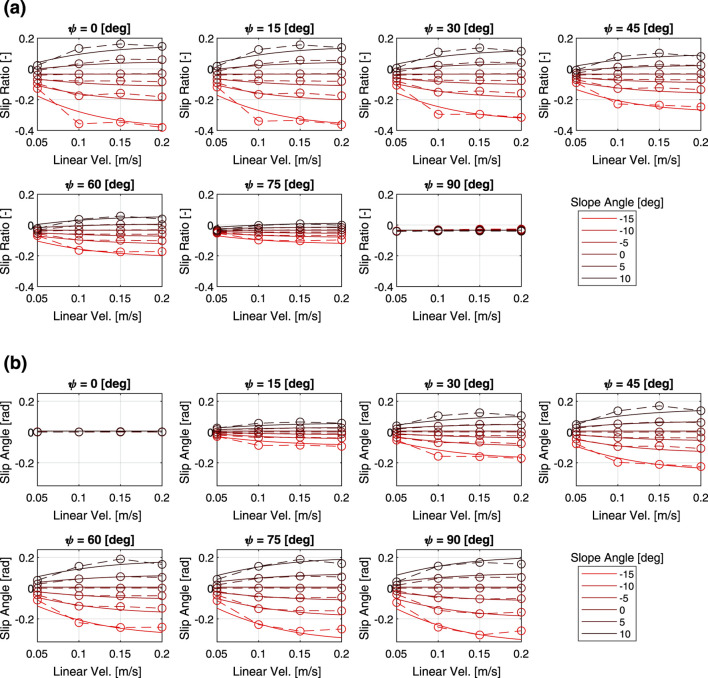
Model fitting results (solid lines: proposed parametric model; dotted lines: simulation data): **(a)** slip ratio vs. linear velocity, **(b)** slip angle vs. linear velocity.

**FIGURE 7:**
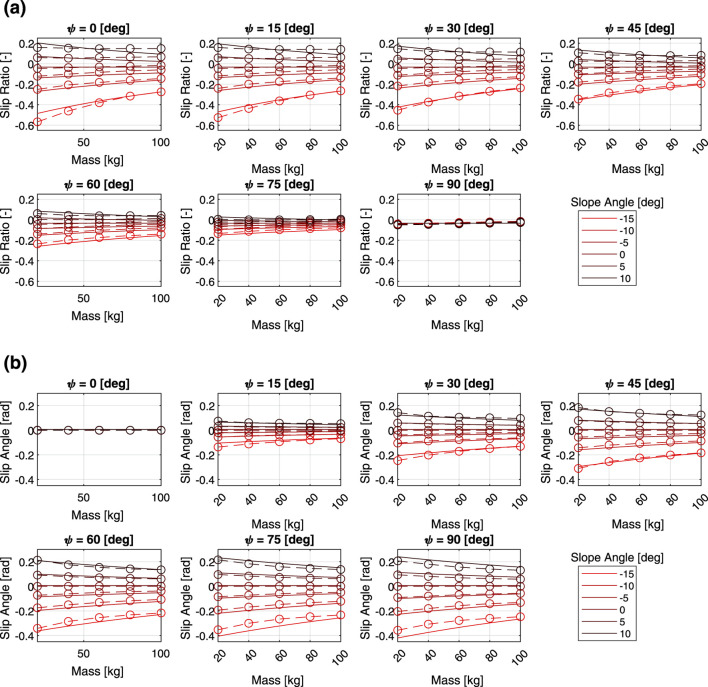
Model fitting results (solid lines: proposed parametric model; dotted lines: simulation data): **(a)** slip ratio vs. total mass, **(b)** slip angle vs. total mass.

The functions $s_{\text{SCM}}$ and $\beta_{\text{SCM}}$ correspond to values contained in the simulation dataset. The values of the hyperparameters obtained from Equations 9, 10 are shown in Table 2. For the optimization algorithm, we used Sequential Quadratic Programming (SQP). The error between the parametric model and the simulation data was evaluated using the Root Mean Squared Error (RMSE). As a result, the RMSE for the slip ratio was 0.0293, and the RMSE for the slip angle was 0.0279 radians. Figure 6 shows the results of comparing parametric slip models and simulations with fixed mass parameters. The primary purpose of calculating RMSE here is to provide reference values when setting the modeling errors in the traversability assessment model, rather than to evaluate the model’s goodness of fit. On the other hand, a previous study examining the relationship between slip ratio, slip angle, and wheel-generated drawbar pull reported that, despite estimation errors, the resulting drawbar pull remains small and does not exhibit large variations Ishigami et al. (2007). The dashed lines represent the simulation results, while the solid lines indicate the parametric model. It was confirmed that the proposed model is expressive enough to effectively reproduce the simulation results. Figure 7 is a graph comparing the slip model and simulation results with constant linear velocity. Similar to the previous results, it can be seen that the proposed model shows similar characteristics to the multi-body dynamics simulation.

**TABLE 2:** Hyperparameter optimization results.

Values of i	1	2	3	4	5
$a_{i}$	0.3988	3.6476	1.4396	1.5659	− 0.0335
$b_{i}$	0.4255	− 3.3654	1.3634	1.1243	− 0.0065

## 3 Traversability assessment model

**FIGURE 8:**
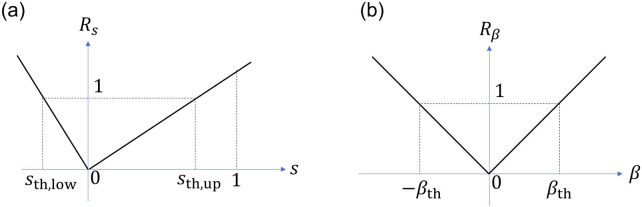
Normalization process. **(a)** Risk factor $R_{s}$ and the slip ratio s , **(b)** the risk factor $R_{\beta }$ and the slip angle β .

In this section, we construct a traversability assessment model that serves as a guideline for rover motion planning by utilizing the proposed parametric model of slip characteristics. By referring to this assessment model during motion planning, the rover is expected to avoid the risk of the vehicle getting stuck and achieve safe traversal toward its target location. Furthermore, since the model adopts a parametric form, it also offers implementation advantages in terms of computational efficiency on onboard processors. Since slip ratio and slip angle represent different concepts, they are nondimensionalized to convert risk factor. When s<0 , the actual translational velocity exceeds the commanded velocity, indicating that the rover is slipping in the direction of travel. Conversely, s=1 represents a state where the wheels are completely stuck, while s>1 indicates that the rover is moving backward. Since positive and negative values of s correspond to different slip state, separate threshold values should be defined to determine hazardous slip conditions in each case. Thus, the risk factor $R_{s}$ for slip ratio is computed as follows:

$$R_{s}=\left\{\begin{array}{cc} |s/s_{\text{th,up}}|\; & if\;s\geq 0,\\ |s/s_{\text{th,low}}|\; & if\;x<0, \end{array}\right.$$ (11)

where $s_{\text{th,up}}>0$ and $s_{\text{th,low}}>0$ . The risk factor $R_{\beta }$ for slip angle is written by

$$R_{\beta }=|\beta /\beta_{\text{th}}|,$$ (12)

where $\beta_{\text{th}}>0$ . Figure 8 shows the normalization process of $R_{s}$ and $R_{\beta }$ , as defined in Equations 11, 12. $s_{\text{th,low}}$ , $s_{\text{th,up}}$ and $\beta_{\text{th}}$ are design parameters and are set to values so that each risk factor exceeds 1 when the slip ratio and slip angle exceed the mission tolerance.

To account for uncertainties, such as modeling errors, each risk factor is treated as a stochastic variable. The risk associated with the slip ratio is assumed to follow the distribution $R_{s}∼N\left(R_{s},\sigma_{s}^{2}\right)$ . Similarly, the risk with respect to the slip angle is assumed to follow $R_{\beta }∼N\left(R_{\beta },\sigma_{\beta }^{2}\right)$ . The conversion from random variables to risk metrics uses Conditional Value-at-Risk (CVaR) (Majumdar and Pavone, 2020; Dixit et al., 2024). CVaR represents the expected value of a random variable when it exceeds a certain threshold. When R follows a normal distribution, CVaR can be obtained analytically as follows:

$$\rho \left(R\right)=\mu +\sigma \frac{\phi \left(\Phi^{-1}\left(\alpha \right)\right)}{1-\alpha },$$ (13)

where ϕ and Φ is the probability density function and cumulative distribution function of a normal distribution, respectively. $\alpha \in \left.\left(0,1\right.\right]$ is the risk level and the larger the value, the more conservative the risk metric. Based on Equation 13, $\rho \left(R_{s}\right)$ and $\rho \left(R_{\beta }\right)$ can be calculated, and the risk is determined by whether each value exceeds 1.

## 4 Performance verification

### 4.1 Robot kinematic model

**FIGURE 9:**
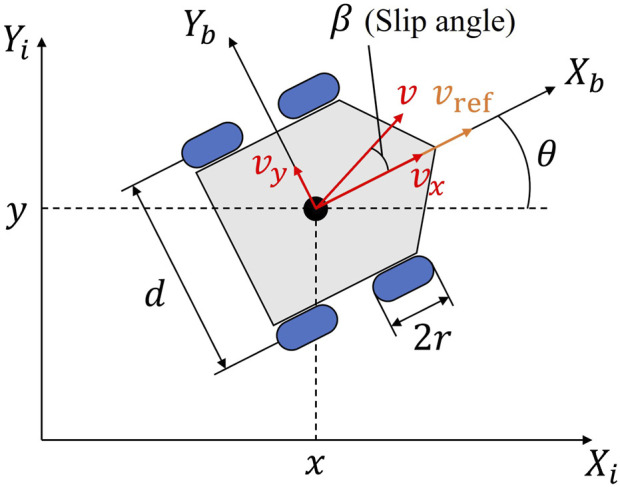
Coordinate frames and rover configuration parameters.

Simulation analysis were conducted to validate the effectiveness of the proposed traversability assessment model. In this study, we assume a differential drive rover as shown in Figure 9. The two-dimensional position and orientation of the rover’s body coordinate system relative to the inertial coordinate system is $x≔{\left[x,y,\theta \right]}^{T}$ , and the rover is assumed to move according to the following kinematics:

$$x=\left[\begin{array}{c} \dot{x}\\ \dot{y}\\ \dot{\theta } \end{array}\right]=\left[\begin{array}{cc} r\cdot \cos\left(\theta \right)/2 & r\cdot \cos\left(\theta \right)/2\\ r\cdot \sin\left(\theta \right)/2 & r\cdot \sin\left(\theta \right)/2\\ -r/d & r/d \end{array}\right]\left[\begin{array}{c} \omega_{l}\\ \omega_{r} \end{array}\right],$$ (14)

where $\omega_{l}$ and $\omega_{r}$ are the angular velocity command values for the left and right wheels, respectively.

The differential two-wheel is a nonholonomic system and has some limitations, which means that the rover cannot move directly sideways and is constrained in the heading direction and the direction of travel. Therefore, in order to ensure that the rover can follow the generated path properly, a motion planning method has to guarantee that the kinematics is satisfied.

### 4.2 Motion planning algorithm

The proposed traversability assessment model should be set as a hard constraint for motion planning because it is a metric for avoiding vehicle getting stuck and any deviation from the specified value may lead to mission failure. As a result, a risk-constrained kinodynamic Rapidly-exploring Random Tree (RRT) algorithm was adopted for performance evaluation in this study. Algorithm 1 represents the algorithm of risk-constrained kinodynamic RRT. The initial state of the rover is $x_{\mathrm{init}}$ and the target state is $x_{\mathrm{goal}}$ . In this case, these state vector consists of position and orientation [x,y,θ] in a 2D plane. The z can be uniquely obtained from terrain elevation data and 2D location. K is the maximum number of iterations. T is the tree. C is a set of parameters used to calculate traversability, such as the mass $m_{0}$ .

Algorithm 1 Risk-Constrained Kinodynamic RRT.

.

1: $T.add\text{\_}vertex\left(x_{\mathrm{init}}\right)$

2: **for** k=1 to K **do**
3: $x_{\mathrm{rand}}\leftarrow \text{Sample}\left(\right)$

4: $x_{\mathrm{near}}\leftarrow \text{NearestVertex}\left(x_{\mathrm{rand}},T\right)$

5: $u_{\mathrm{rand}}\leftarrow \text{Sample}\left(\right)$

6: $x_{\mathrm{new}}\leftarrow \text{Propagation}\left(x_{\mathrm{near}},u_{\mathrm{rand}}\right)$

7: $\rho \left(R_{s}\right)\leftarrow \text{SlipRatioRisk}\left(x_{\mathrm{new}},u_{\mathrm{rand}},C\right)$

8: $\rho \left(R_{\beta }\right)\leftarrow \text{SlipAngleRisk}\left(x_{\mathrm{new}},u_{\mathrm{rand}},C\right)$

9:  **if** $\rho \left(R_{s}\right)<1$ **and** $\rho \left(R_{\beta }\right)<1$ **then**
10: $T.add\text{\_}vertex\left(x_{\mathrm{new}}\right)$

11: $T.add\text{\_}edge\left(x_{\mathrm{near}},x_{\mathrm{new}}\right)$

12:   **if** $distance\left(x_{\mathrm{new}},x_{\mathrm{goal}}\right)<\epsilon$ **then**
13:     **return** T

14:    **end** **if**
15:  **end** **if**
16: **end** **for**
17: **return** T

The algorithm is an improvement of the basic RRT (LaValle, 1998) to handle both kinematic constraints and traversability constraints (LaValle and Kuffner, 2001). The exploration starts with the tree initialized with the rover’s initial state $x_{\mathrm{init}}$ . In each iteration, a random sample of the 2D position and orientation $x_{\mathrm{rand}}$ is selected from the exploration space (line 3). The function NearestVertex(x,T) searches for the vertex that is closest to the input x (line 4). In this process, relative attitude is not considered; instead, the nearest neighbor is determined by computing the Euclidean distance based on relative position. $u_{\mathrm{rand}}$ is a 2D state variable consisting of the rotational velocities of the rover’s wheels. A value is randomly sampled from the possible range of angular velocities (line 5). Propagation(x,u) on line 6 is a function to compute the kinematics based on the state x and control input u using Equation 14. Substituting $x_{\mathrm{near}}$ and $u_{\mathrm{rand}}$ into the function, the rover’s state at the next time step is calculated and stored as $x_{\mathrm{new}}$ . Instead of directly sampling the state variables and adding them to the tree, the control inputs are sampled and propagated through time, ensuring that the generated random state variables are dynamically and kinematically valid. The risks based on the generated state $x_{\mathrm{new}}$ , control input $u_{\mathrm{rand}}$ , and parameter C are calculated (lines 7–8). If the risks exceed a threshold, the state is rejected as it presents a high risk of slip. Otherwise, the new vertex and edge information is added to the tree T . Since the algorithm is sampling-based method, it easily handles constraints by simply checking if the new state satisfies the constraints. If $x_{\mathrm{new}}$ is sufficiently close to the goal, the exploration is terminated; otherwise, it continues (lines 9–15).

In this study, the effectiveness of the traversability metric is validated using a risk-constrained kinodynamic RRT. The primary objective of the simulation is to evaluate the performance of the modeling method; therefore, a basic RRT was intentionally used as the motion planning algorithm. Improvements to the motion planning algorithm are considered beyond the scope for this work. Note that the proposed risk metric is not limited to this approach and can be applied to other motion planning techniques as well. Numerous constraint-aware motion planning methods exist, and they can be flexibly selected based on the specific application.

### 4.3 Simulation results

**FIGURE 10:**
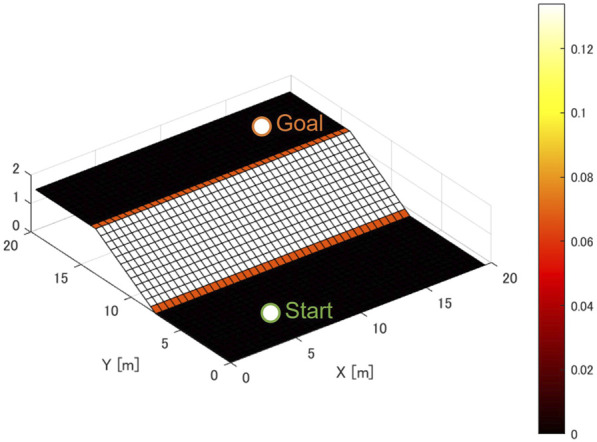
Terrain for motion planning simulations.

The terrain map used for validation is shown in Figure 10. The starting point is set at [x,y]=[10,3] and the goal point at [x,y]=[10,17] . The rover is assumed to initially face the slope direction, ψ=0 . The heading angle of the rover at the goal point is not specified. Both points are placed on a flat surface with no slope. The slope between the starting point and the goal point was set to an inclination of 15°. When torque limitations are present, slopes beyond a certain angle cannot be climbed, which can affect operational strategies. However, torque limitations are hardware constraints of the rover itself and are not directly effected to the slip estimation model or the traversability assessment model proposed in this study. Therefore, the effects of torque limitations are not considered in this simulation. In addition, performance comparisons on actual hardware are out of scope in this paper and the feasibility of implementing the algorithm on a space-grade CPU is not also evaluated.

**FIGURE 11:**
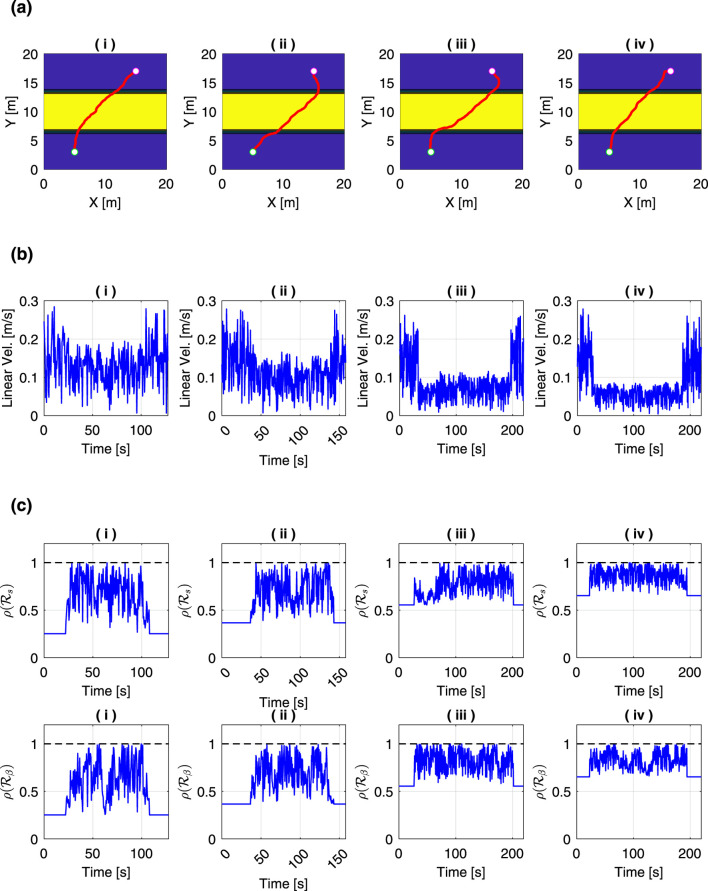
Comparison of motion planning results under different risk levels α : **(a)** generated paths, **(b)** time history of the linear velocity, and **(c)** time history of the risk metrics. The risk levels were set to (i) α=0.5 , (ii) α=0.7 , (iii) α=0.9 , and (iv) α=0.95 .

Figure 11a shows a graph comparing how the generated paths change depending on the risk level. In this analysis, wheel radius r=0.168 m, the distance between wheels d=0.5 m, and mass m=50 kg were assumed. The hyperparameters for slip ratio and slip angle were obtained through optimization calculations, using the values from Table 2. The parameter settings for the traversability assessment model are shown in Table 3. The risk levels for slip ratio and slip angle, denoted by α , are set to the same values, and in Figure 11a, they are set to (i) 0.5, (ii) 0.7, (iii) 0.9, and (iv) 0.95, respectively. By adopting the Kinodynamic RRT, smooth paths that satisfy the kinematic constraints were successfully generated. In all conditions, paths were generated that climbed diagonally in sloped areas. The risk metric for slip ratio decreases as the heading angle increases when climbing the slope, whereas the risk metric for slip angle decreases as the heading angle decreases, as shown in Equations 3, 4. Therefore, it can be considered that the path climbs the slope at a balanced angle that reduces both risk metrics.

**TABLE 3:** Parameter settings for traversability assessment model.

Parameter	Value
Upper threshold $s_{\text{th,up}}$	0.1
Lower threshold $s_{\text{th,low}}$	− 0.1
Variance $\sigma_{s}$	0.1
Threshold $\beta_{\text{th}}$	0.1
Variance $\sigma_{\beta }$	0.1

The comparison of the time history of the rover’s linear velocity is shown in Figure 11b. During periods when the rover is not climbing a slope, the linear velocity reaches nearly 0.3 m/s, but it decreases during slope climbing. Specifically, in Figure 11b, i the velocity during climbing remains below 0.2 m/s, while in Figure 11b, iv, it decreases to below 0.1 m/s. Thus, it was confirmed that the linear velocity during slope climbing tends to become lower as the value of α increases. Equations 3, 4 show that both the slip ratio and slip angle risk metrics decrease as the linear velocity decreases. These results demonstrate that the conservativeness of the motion planning can be adjusted by the risk level α .

Figure 11c is the comparison of the time history of the risk metrics. From this figure, we can observe that the both risks of slip ratio and slip angle increased during the slope-climbing phase, and by reducing the linear velocity, the rover was able to adjust the risks to avoid exceeding the threshold of 1 while still moving. Even when the rover is located in an area where the slope angle is 0°, $\rho \left(R_{s}\right)$ and $\rho \left(R_{\beta }\right)$ were not zero, indicating that a positive bias exists. This bias is caused by the second term in Equation 13, and it becomes larger as the risk level α increases. This explains why the conservativeness of the motion planning varies depending on the value of α .

**FIGURE 12:**
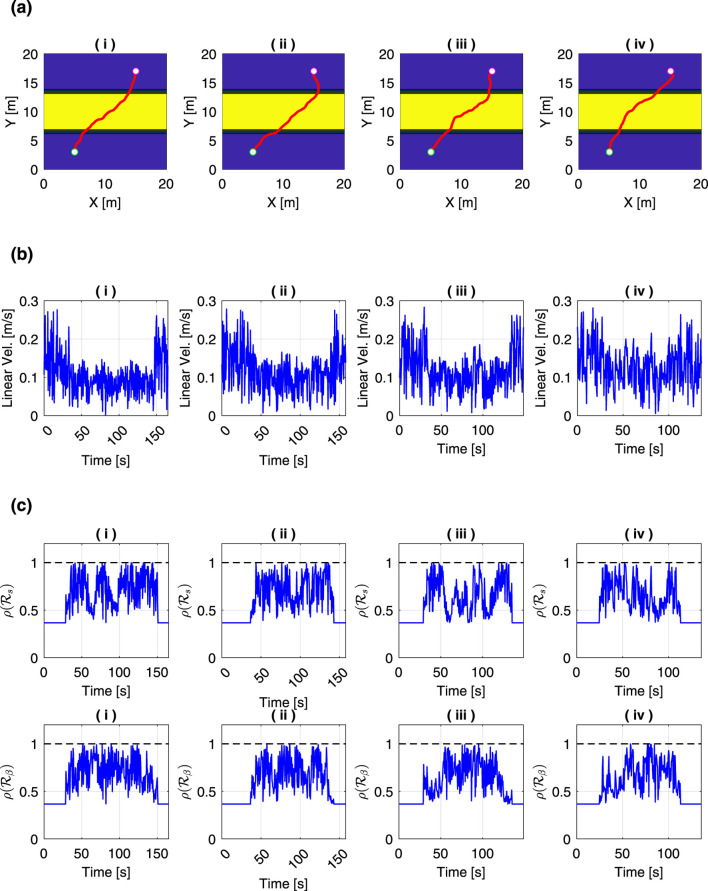
Comparison of motion planning results under different total mass m : **(a)** generated paths, **(b)** time history of the linear velocity, and **(c)** time history of the risk metrics. The total masses were set to (i) m=30 kg, (ii) m=50 kg, (iii) m=70 kg, and (iv) m=90 kg.

Next, we evaluated how the motion planning results varied with mass. Figure 12a shows the generated paths compared by different masses. The hyperparameters of the slip estimation model were set based on the values listed in Table 2. Parameters for the traversability assessment model were taken from Table 3, and the risk levels α for both slip ratio and slip angle were set to 0.7. Regarding the variances, they were set to include a margin, considering the modeling errors of the slip estimation model: the RMSE of slip ratio was 0.0293, and the RMSE of slip angle was 0.0279. In all cases, the generated path tended to climb the slope diagonally. In Figures 12a iii, iv there are sections where the path climbs more directly uphill.

This is because the increased mass results in a lower slip ratio, as described by Equation 3, which makes it less risky to move more directly in the slope direction.

The comparison of the time history of the rover’s linear velocity is shown in Figure 12b. As the mass increases, there is a tendency for the linear velocity while climbing a slope to become faster. Both the slip ratio and slip angle decrease with increasing mass, as shown in Equations 3, 4. Conversely, higher linear velocity tend to increase the slip ratio and slip angle. In other words, increasing the mass reduces the risk of slipping, which allows for higher linear velocity during slope climbing.

Figure 12c shows the time history of the risk metric for each case. The risk metrics remained below 1 in all cases. It was demonstrated that by using the RRT algorithm, the rover can safely reach the target position even under conditions of increased slip risk due to mass parameter settings, by appropriately adjusting its linear velocity and trajectory.

## 5 Conclusion

In this study, we first proposed parametric models of slip ratio and slip angle, which use four input parameters: slope angle, heading angle, mass, and wheel angular velocity. By comparing the results with multi-body dynamics simulations, it was confirmed that the proposed model has sufficient expressive power to estimate slip. We also proposed a traversability assessment model that incorporates the parametric models for slip ratio and slip angle. This assessment model is designed to allow tuning, taking into account uncertainties such as modeling errors. To evaluate the effectiveness of the proposed method, we conducted a simulation evaluation. As an example of a motion planning method, we introduced the risk-constrained kinodynamic RRT algorithm, which incorporates the proposed assessment model. Through the simulation results, we analyzed how the coefficients of the slip parametric model affect the generated paths. For future work, it would be useful to verify the effectiveness of the proposed slip estimation model through real-world tests. The test results would be an important step toward practical application. Additionally, we intend to model the estimation errors of the four input parameters when calculated onboard and evaluate their impact on the robustness of the motion planning approach.

## Data Availability

The original contributions presented in the study are included in the article/supplementary material, further inquiries can be directed to the corresponding author.
